# Management of a young female patient with Fournier's gangrene and Lemierre's syndrome

**DOI:** 10.11604/pamj.2014.18.275.4602

**Published:** 2014-08-04

**Authors:** Theodoros Aslanidis, Athena Myrou, Maria Giannakou-Peftoulidou

**Affiliations:** 1 Intensive Care Unit, Department of Anesthesia and Intensive care, “A.H.E.P.A” University Hospital, 1 St. Thessaloniki, Greece

**Keywords:** Fournier's gangrene, Lemierre's syndrome

## Abstract

Fournier's gangrene is an acute, rapidly progressive, and potentially fatal, infective necrotizing fasciitis affecting the external genitalia, perineal or perianal regions. Lemierre's syndrome is a condition characterized by thrombophlebitis of the internal jugular vein and bacteremia caused by primarily anaerobic organisms, following a recent oropharyngeal infection. Although the literature about either of them is rich, there is no report about co-appearance of the two syndromes. We present the case of a young healthy female patient who suffered concomitantly from Fournier's gangrene and Lemierre's syndrome after minor surgery.

## Introduction

Fournier's gangrene, first reported in 1883, is an acute, rapidly progressive, and potentially fatal, infective necrotizing fasciitis affecting the external genitalia, perineal or perianal regions, which commonly affects men. Women and children, not mentioned in the original report, are also known to suffer from it. However, reports of women remain scanty. There has been an increase in number of cases in both sexes in recent times. But despite advanced management, mortality is still high and averages 20-30%. Lemierre's syndrome, a condition characterized by thrombophlebitis of the internal jugular vein and bacteremia caused by primarily anaerobic organisms, following a recent oropharyngeal infection, was also referred by some authors during 1980s and 1990s as “forgotten disease”. Since the late 1990s, for reasons that are not clear, there has been an increase in the reporting of Lemierre's syndrome. Today, there are a lot of reports for either syndrome. We present the case of a young female patient who suffered concomitantly from Fournier's gangrene and Lemierre’ syndrome. To our knowledge, this is the first report of co-appearance of both of them in the same patient.

## Patient and observation

**Figure 1:**
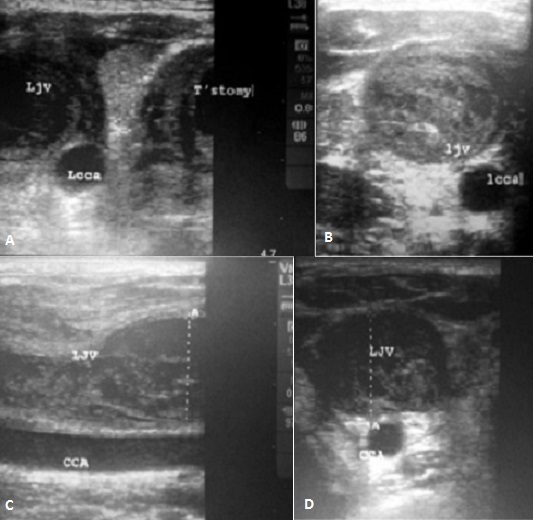
Vascular ultrasound examination of Left jugular vein. (A) and (B) At diagnosis. Thrombus can be seen down to the level of tracheotomy (C) and (D) At 6th day of therapy. Partially resolving of the occlusion

A 23 year old female patient (weight 67kg, height 171cm, BMI 22.91kg/cm^2^) presented to emergency department with fever (39.4°C), tachycardia (116 bpm), hypotension (95/45 mmHg), tachypnoea (25 breaths/min), sore throat, neck pain, diffuse pain and tissue oedema in the right-side gluteal region. She was appointed to hospital by a private outpatient surgical center where she had undergone perineal abscess drainage a week before. Recent medical history included also endometrial curettage for ERPC a month before and conservative therapy for hemorrhoids 15 days after. Past medical history included Henoch-Schönlein purpura during childhood. Social history included smoking (11 pack-years), family history was non-contributory. She had no known drug allergies. Complete blood count revealed marked neutrophilic leukocytosis (WBC 23.400 /lt). A CT scan revealed small bilateral pleural effusions, small hepatic enlargement, Gerota's fascia thickening, perineal soft tissue stranding with fascial thickening and small bubbles of air. Fournier's gangrene was set as working diagnosis (calculated FGSI 8, UFGSI 10 and LRINEC 7) and the patient was transported to operation room for immediate radical surgical debridement of necrotic tissue. Blood and surgical material was taken for microbiologic analysis. Due to the extent of the surgical debridement, the patient was admitted postoperatively to the ICU (SOFA score 6, APACHE II score 12); where sedation (midazolam 30 mcg/kg/hr c.iv), analgesia (fentanyl 3 mcg/kg/min c.iv), triple antibiotic regimen with meropenem 2gr q8h i.v., clindamycin 600mg q 6h i.v. and daptomycin 500mg q.d. i.v., gastroprotective and antithrombotic prophylaxis were initiated. During the next 3 days a second CT and an obstetrical consultation were conducted with no significant findings. Human immunodeficiency virus and diabetic screen were also negative. Yet, the patient remained febrile. Microbiology cultures from material taken from the surgical trauma area revealed *Candida albicans, Staphylococcus epidermidis and Klebsiella pneumonia*; hence antibiotic regimen was modified and antimycotic treatment (caspofungin 100mg q.d. i.v.) was initiated too. Debridements were repeated every 24 hours but due to the extent of the lesions, on the 10^th^ day, the patient was transported to operation room for temporary ileostomy till the end of the therapy. Percutaneous tracheotomy was also performed. Hyperbaric oxygen therapy was not available at the setting. One day after the second surgery, patient's condition complicated with MRSA ventilation associated pneumonia. Along with that, a localized oedema appeared in her left upper limb. A palpable mass along the anterior margin of the sternocleidomastoid muscle was also noticed. Vascular ultrasound examination revealed occlusion of left internal jugular vein (Figure 1). Transcranial Doppler examination was normal. Dental consultation and examination of soft palate and peritonsilar tissues showed no abnormalities. Linezolide 600 mg q12h i.v. was added to antibiotic regimen. Since debridements were going on, simultaneous anticoagulation therapy was started with only bemiparine 2500 b.i.d. s.c. Gradually, the patient became afebrile and her clinical condition improved. She was discharged from ICU 13 days later. A follow- up CTA confirmed the disappearance of the left jugular vein thrombosis. About a month after her ICU discharge, an operation was performed for ileostomy closure and reconstruction of the right gluteal region. On a follow-up examination 6 months later, the young woman had returned to her normal everyday life.

## Discussion

Necrotizing fasciitis, a life-threatening rare infection of the soft tissues, is a medical and surgical emergency. It can be divided into 4 types: polymicrobial, often bowel flora derived (I), monomicrobial, skin or throat derived (II), Gram-negative monomicrobial (III) and fungal (IV)[1, 2]. Fournier's gangrene (type I) true incidence of is unknown. A retrospective case review revealed 1726 cases documented in the literature from 1950 to1999[3]. An average of 97 cases per year was reported from 1989 to 1998. The overall frequency reported is 1:7500, most cases occur between 30-60 years of age and male-to female ration in large series is about 10:1[3]. It is interesting that ICD-9 code for Fournier's gangrene (608.83) is found under the diseases of the male genital organs subheading (no diagnostic code for females)[4]. In women, septic abortions, vulva or Bartholin gland abscesses, hysterectomy, episiotomy and anorectal infections are documented as etiological sources. And though Fournier gangrene follows the same pattern as in men, female patients receive more often fecal diversion[5–7]. Lemierre's syndrome's incidence is between 0.6 and 2.3 per million. Despite modern antibiotic availability, mortality rates are between 4% and 18%[8]. *Fusobacterium necrophorum* is the most common pathogen reported, but several other microorganisms have been also implicated alone or in combination with *Fusobacterium necrophorum*, including *Bacteroides species*, groups B and C *streptococci, Streptococcus oralis, Fusobacterium nucleatum, Streptococcus intermedious, Staphylococcus epidermidis, Enterococcus species, Proteus mirabilis and Arcanobacterium haemolyticum* [9]. To our knowledge, this is the first report of a case with concomitant presence of these two syndromes; and it should not be considered as a Meleney′s synergistic gangrene case (a synergistic interaction between microaerophilic nonhemolytic streptococci and aerobic hemolytic staphylococci which produces extensive tissue necrosis with ulceration).

## Conclusion

The apparent lower reported incidence of Fournier's gangrene in women is by no means a reason to underestimate this condition. Lemierre's syndrome on the other hand often can present a diagnostic challenge. Clinicians need to be aware of both syndromes as the initial symptoms can be followed by a fulminant course. Aggressive and timely proper treatment is vital as anything (as in this case co-occurrence of both syndromes in an otherwise healthy person) can happen.
